# Cracking modes and force dynamics in the insertion of neural probes into hydrogel brain phantom

**DOI:** 10.1088/1741-2552/ad5937

**Published:** 2024-07-05

**Authors:** Gen Li, Dongyeol Jang, Yieljae Shin, Yi Qiang, Yongli Qi, Shuodao Wang, Hui Fang

**Affiliations:** 1 Thayer School of Engineering, Dartmouth College, Hanover, NH 03755, United States of America; 2 School of Mechanical & Aerospace Engineering, Oklahoma State University, Stillwater, OK 74078, United States of America

**Keywords:** insertion dynamics, neural probe, hydrogel brain phantom, cracking modes

## Abstract

*Objective*. The insertion of penetrating neural probes into the brain is crucial for advancing neuroscience, yet it involves various inherent risks. Prototype probes are typically inserted into hydrogel-based brain phantoms and the mechanical responses are analyzed in order to inform the insertion mechanics during *in vivo* implantation. However, the underlying mechanism of the insertion dynamics of neural probes in hydrogel brain phantoms, particularly the phenomenon of cracking, remains insufficiently understood. This knowledge gap leads to misinterpretations and discrepancies when comparing results obtained from phantom studies to those observed under the *in vivo* conditions. This study aims to elucidate the impact of probe sharpness and dimensions on the cracking mechanisms and insertion dynamics characterized during the insertion of probes in hydrogel phantoms. *Approach*. The insertion of dummy probes with different shank shapes defined by the tip angle, width, and thickness is systematically studied. The insertion-induced cracks in the transparent hydrogel were accentuated by an immiscible dye, tracked by *in situ* imaging, and the corresponding insertion force was recorded. Three-dimensional finite element analysis models were developed to obtain the contact stress between the probe tip and the phantom. *Main results*. The findings reveal a dual pattern: for sharp, slender probes, the insertion forces remain consistently low during the insertion process, owing to continuously propagating straight cracks that align with the insertion direction. In contrast, blunt, thick probes induce large forces that increase rapidly with escalating insertion depth, mainly due to the formation of branched crack with a conical cracking surface, and the subsequent internal compression. This interpretation challenges the traditional understanding that neglects the difference in the cracking modes and regards increased frictional force as the sole factor contributing to higher insertion forces. The critical probe sharpness factors separating straight and branched cracking is identified experimentally, and a preliminary explanation of the transition between the two cracking modes is derived from three-dimensional finite element analysis. *Significance*. This study presents, for the first time, the mechanism underlying two distinct cracking modes during the insertion of neural probes into hydrogel brain phantoms. The correlations between the cracking modes and the insertion force dynamics, as well as the effects of the probe sharpness were established, offering insights into the design of neural probes via phantom studies and informing future investigations into cracking phenomena in brain tissue during probe implantations.

## List of abbreviations

**Table:** 

Abbreviations	Definition	Unit
*F*	Insertion force	mN
$\delta $	Displacement	mm
*α*	Tip angle	°
*W*	Shank width	*μ*m
*T*	Shank thickness	*μ*m
${\delta _d}$	Dimpling	mm
${F_p}$	Puncture force	mN
${F_e}$	Ending force	mN
${K_i}$	Pre-puncture stiffness	mN mm^−1^
${K_e}$	Post-puncture stiffness	mN mm^−1^
*L_c_*	Characteristic tip width	*μ*m
${\delta _a}$	Actual probe penetration depth	mm
${\delta _p}$	post-contact probe downward displacement	mm
${\delta _c}$	the depth of the cracked or damaged region	mm
${\sigma _v}$	von Mises stress	kPa
$\phi $	Ratio of post-puncture stiffness to pre-puncture stiffness	Dimensionless
${F_{{\text{compression}}}}$	Compression force	mN
${F_{{\text{friction}}}}$	Friction force	mN
${F_{{\text{adhesion}}}}$	Adhesion force	mN
*A*	Actual contact area	${{\mu }}{{\text{m}}^2}$

## 1. Introduction

The evolution of neural interfaces has significantly advanced the understanding of the brain, enabling a wide range of applications spanning fundamental neuroscience studies to clinical utilization. While penetrating probes offer direct interfaces with unparalleled proximity to brain tissue and neurons that enable precise electrical activity recording or stimulation at the single-cell level, their insertion poses risks of probe deformation, brain trauma, and tissue foreign body response [1–3]. Current neural probe designs are continuously evolving with variations in functions, footprints, and materials (*e.g.* polymers, metals, and silicon) [4–7]. The development of prototype neural probes requires meticulous tests and validations to mitigate risks such as mechanical failures associated with buckling or fracture of the probes, especially when smaller probe footprints are desirable [8]. As it is impractical to rely solely on *in vivo* tests during the entire probe design process, insertion tests utilizing brain-mimicking phantoms have been developed [9]. In particular, hydrogels that retain a large water content within a 3D crosslinked network have been used as phantoms mimicking the properties of different biological tissues [10]. Agarose with 0.4%–0.6% w/w water content is commonly employed as a brain tissue mimic due to its similar Young’s modulus (ranging between 1 and 10 kPa) to brain, cost-effectiveness, and straightforward preparation [11]. For example, it is used in insertion tests of various penetrating probes and arrays [12–18] to characterize insertion dynamics and to visualize probe status leveraging the hydrogel’s transparency.

The characterization of insertion dynamics, commonly represented by force-time (*F-t*) and force-displacement (*F-δ*) curves, has been extensively utilized in the investigations of neural probe insertion. In summary, the key factors that influenced the neural probe included three categories: (1) the geometry (footprint and sharpness) of the neural probe, (2) the material interface properties, and (3) the insertion conditions. A smaller force was, in general, used as an indication or criterion for less damage. For the effect of probe footprint and sharpness, for example, Sharp *et al* [19]. Obaid *et al* [20] and Perna *et al* [21] have shown that the insertion force decreases with a decrease in probe cross-sectional area, and a sharpened tip typically leads to lower penetration force from *in vivo* experiments tested with stainless steel probe, tungsten microwire, and silicon probe. Joo *et al* [22] has achieved a 3D sharpened silicon shuttle that can inserted through the dura with minimal tissue compression. Han *et al* [23] has precisely controlled the tip shape of silicon probe also in 3D using deep reactive ion etching and shown the reduction of insertion force in both brain and spinal cord. As the field progresses, the current popular neural probes have been configured with miniaturized footprints and sharper tips, as reviewed in table S1. For the material interface properties, the main efforts were to reduce the friction or adhesion force between the probe and tissue. For example, Lee *et al* [13] demonstrated the lubricant-coated probe, which lowers the friction force and thus reduces the insertion damage. Middya *et al* [24] has coated the microwire with hydrophobic PDMS to lower the friction with the surrounding tissue. Regarding the insertion conditions, insertion speed has been widely investigated with a wide range from *µ*m s^−1^ to m s^−1^ but not fully conclusive. Casanova *et al* [25] have investigated the *in vivo* force at varying insertion speeds (2–10 mm s^−1^) for needles and declaimed that the friction stress decreased with increasing the insertion speed. The pneumatical insertion (8.3 m s^−1^) has been used as the most effective method for the Utah array without excessive tissue trauma [26]. However, Fiáth *et al* [27] declaimed that slow insertion (2 *µ*m s^−1^) of silicon probe can improve the quality of the acute recording. Since the conclusion is debatable, we have included table S2 in the supplementary information, reviewing some of the available literature and their key conclusion. Moreover, recent advancements using ultrasonically actuated insertion have also been implemented for trauma and inflammation reduction, as reported by Chen *et al* [17].

While these studies extensively analyzed various aspects, such as the origins of the forces, the dimpling and puncture force dependency, and empirical fitted scaling laws for force versus the probe size, a large gap remains in understanding the fundamental cracking mechanisms during the insertion process and studies addressing insertion-induced cracks remain limited. It is crucial to emphasize the extreme challenge of simultaneously measuring ultrasensitive forces *in vivo* while visualizing microcracks or damage in opaque brain tissue during microscale neural probe implantation. This necessitates the use of transparent mimicking media such as hydrogel phantoms. Additionally, even though hydrogel phantoms are less heterogeneous than brain tissue, they possess similar nonlinear hyperelastic behavior as in tissues, which will significantly affect the cracking phenomena within them. Recently, the emergence of super-shear cracks in brittle hydrogels induced by tensile forces has challenged classical fracture mechanics, reshaping the fundamental understanding of fracture dynamics [28, 29]. In neural engineering, the emergence of cracks and fractures during insertion procedures, particularly those leading to vasculature disruption, poses significant risks that could potentially lead to the cessation of electrophysiological responses and profound chronic immune reactions [30]. Different groups have utilized the insertion dynamics as an indirect gauge for identifying damages [31–33], without any explicit measurement of the real damage and cracks. Filling this knowledge gap in cracking mechanisms is crucial for accurately analyzing and interpreting the probe insertion dynamics. We believe the systematical analysis of cracking phoneme in transparent hydrogel brain phantom is the first step, and the new insights and understanding obtained from the gel insertion test will eventually translate into the understanding of the implantation process and associated damage in brain tissue.

In this study, the insertion of shank-shaped polymer neural probe dummies (with sizes relevant to contemporary neural probes) into 0.6% w/w agarose hydrogel brain phantoms was systematically studied via *in situ* force-displacement recordings and imaging of the corresponding crack formation and propagation process. The main focus is to elucidate the impact of the neural probe geometry (tip angle, shank width, and thickness) on the cracking modes and insertion dynamics. In addition, preliminary investigations on the effect of different insertion speed and different probe material interface were also discussed. For the first time, experimental observations unveiled two distinct cracking modes in hydrogel phantoms during insertion tests, each leading to a unique type of force dynamics. The results and discussions highlight the importance of accounting for cracking mechanisms in phantom studies and considering fracture when evaluating probe insertion in phantoms and tissue.

## 2. Methods

### 2.1. Fabrication of probe dummies with different geometry

**Figure 1.**
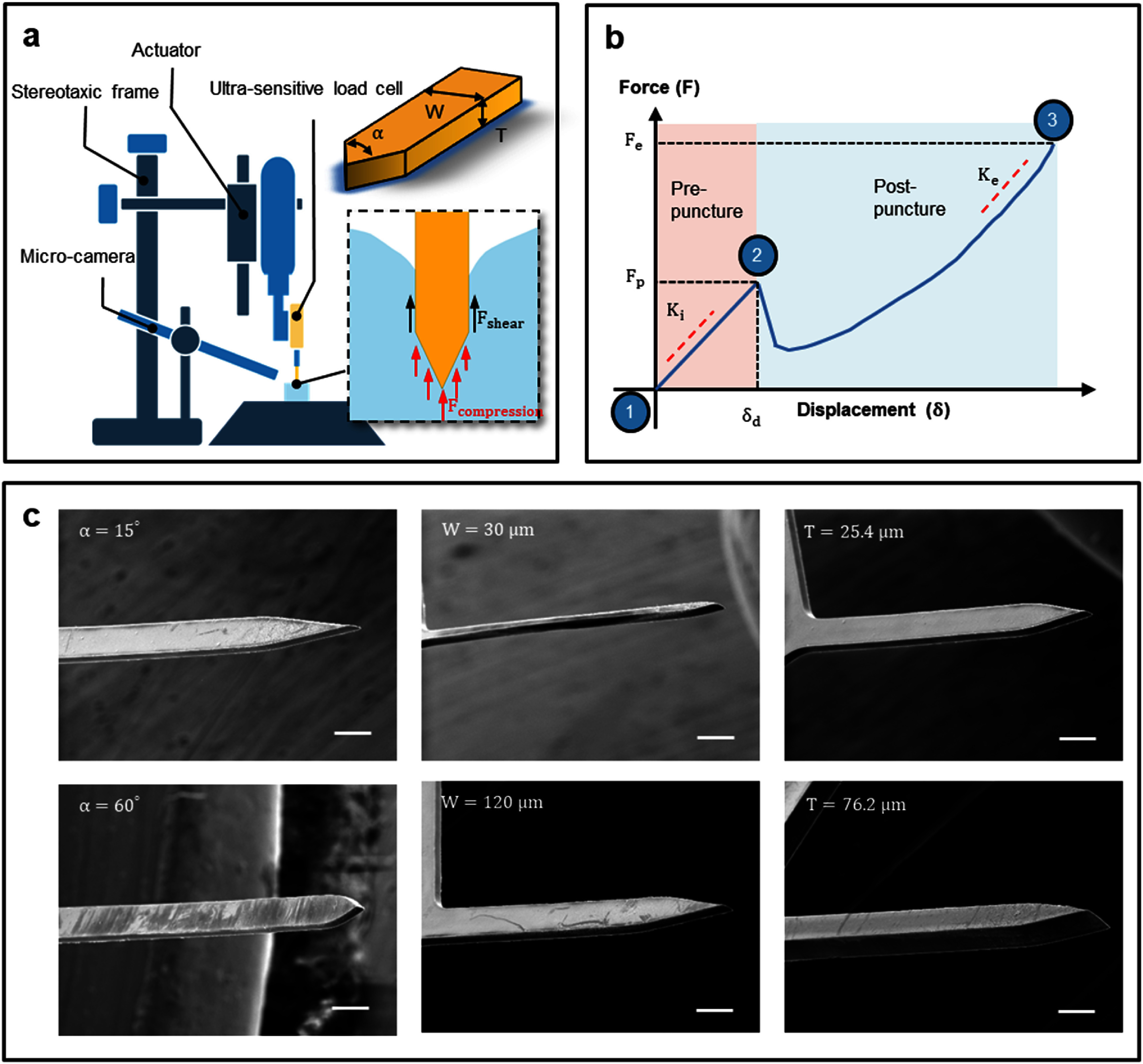
Measurement of insertion force as a function of probe geometry. (a) Setup for measuring insertion force with parameters defining typical probes (*α, W*, and *T*) and a depiction of the force comprising shear and compression components. (b) An illustrative typical *F-δ* curve for neural probes inserted into a brain phantom. The important characterizations of the force dynamics were listed here: dimpling (${\delta _d}$), puncture force (${F_p}$); ending force (${F_e}$); pre-puncture stiffness (${K_i}$); post-puncture stiffness (${K_e}$). (c) SEM images showcasing shank-shaped probes with typical dimensions. Scale bar: 100 *μ*m.

Mechanical probe dummies were fabricated using Kapton to systematically investigate the insertion dynamics of neural probes in the brain phantom as a function of their geometry (figure 1). Specifically, single-shank probes of different tip angles (*α*), shank widths (*W*), and thicknesses (*T*) were fabricated using direct laser cutting (LPKF; Protolaser U4) on commercial Kapton films with a thickness of 0.5 mil (12.7 *μ*m) to 3 mil (76.2 *μ*m), as outlined in table 1. The reasons for fabricating the mechanical dummy from DuPont™ Kapton^®^ polyimide films are: (1) it is one of the popular, commercially available substrates for polymer-based neural probes, which has been widely used among different groups due to its reliable thermal, mechanical, and chemical performance. (2) It facilitates the fabrications requiring the tuning of multiple geometrical parameters. The probe length measured from the tip to the base was controlled at 1.7 mm. The representative scanning electron microscope (SEM) images are provided in figure 1(c). A baseline probe geometry was established, featuring $\alpha $ = 20°, *W* = 90 *μ*m, and *T* = 25.4 *μ*m. It is important to emphasize that the baseline probe configuration was carefully selected based on a comprehensive review of popular neural probes available in commercial markets and reported in the literature (table S1). For instance, the configuration of the Neuropixel probe [34] was set as follows: *α* = 20°, *W* = 70 *μ*m, and *T* = 20 *μ*m. Similarly, one of the configurations for a NeuroNexus probe [35] was determined as: *α* = 45°, *W* = 120 *μ*m, and *T* = 15 *μ*m. Besides, to preliminarily test the effect of different materials interface, a special comparison study using tungsten microwires (float tip, diameter of 50 *μ*m, W5607, Advent Research Materials), which has a similar footprint as our baseline probe, was conducted.

**Table 1.** Mechanical probe dummies with different geometry.

*α* (°)	*W* (*μ*m)	*T* (*μ*m)
15, 20, 30, 45, 60, 180	90	25.4
20	30, 60, 90, 120	25.4
20	90	12.7, 25.4, 50.8, 76.2

### 2.2. Fabrication of the brain phantoms

The 0.6% w/w agarose hydrogel (Agarose BP160-100, Fisher Scientific) is utilized in this study as brain phantoms. A customized mold was fabricated via 3D printing to define a cylindrical cavity geometry with a diameter of 12 mm and a height of 8 mm. The gel solution (0.24 g of agarose in 40 g of deionized water) was prepared and heated at 140 °C for 1 h, then delicately injected into the mold using a syringe, allowing meticulous control over the external surface geometry of the gel. The gel was solidified in the mold at room temperature for 2 h. To maintain the gel’s properties and prevent water evaporation, all molded gel samples were securely stored in a humidity-controlled chamber before insertion tests. The effectiveness of the humidity control was assessed and showed in figure S1, demonstrating negligible changes in sample weight compared to those directly exposed to ambient lab environment.

### 2.3. Insertion force measurement in brain phantom tests

The force-displacement response from brain phantom insertion tests is commonly measured for analyzing the insertion dynamics of probes, as illustrated in figure 1(b) which depicts three events: (1) the beginning of contact, detected by the start of non-zero contact force; (2) puncture, featuring a sudden drop in forces from a peak puncture force $\left( {{F_p}} \right)$ corresponding to a displacement $\left( {{\delta _d}} \right)$ commonly referred to as dimpling; (3) the conclusion of the test, marked by the end of the curve with the ending force $\left( {{F_e}} \right)$. At the first stage from status (1) to (2), normally referred to as pre-puncture, the force typically increases linearly until puncture, where the slope ${K_i}$reflects the stiffness of the phantom. Post puncture, the force generally increases again until the end of the insertion process and features a slope of ${K_e}$. It should be noted that the current work is restricted to post-contact probe displacement of up to 1.5 mm, which is selected within the range of the length for typical intracortical probes (1–2 mm) for rodent models [3].

An ultra-sensitive force measurement system is designed to accurately measure the sub-mN level insertion forces in single-shank micro probe insertion tests and the complex variations of force dynamics as described above. A miniature S-Beam load cell (FUTEK, FSH03869) with high resolution (7 *μ*N) USB output (FUTEK, FSH04720) was integrated with the commercial neural implant inserter (NeuralGlider) and combined with a stereotaxic frame, as illustrated in figure 1(a). All insertion trials reported in the main figures utilized a consistent insertion speed of 50 *μ*m s^−1^, with the probe oriented perpendicularly to the contact surface. Besides, a preliminary experiment investigating the effect of insertion speed was conducted and summarized in the Supplementray information. Two extra different insertions speed, namely, 5 *μ*m s^−1^ (slow) and 500 *μ*m s^−1^ (fast) were used.

### 2.4. *In situ* visualization of crack morphology

**Figure 2.**
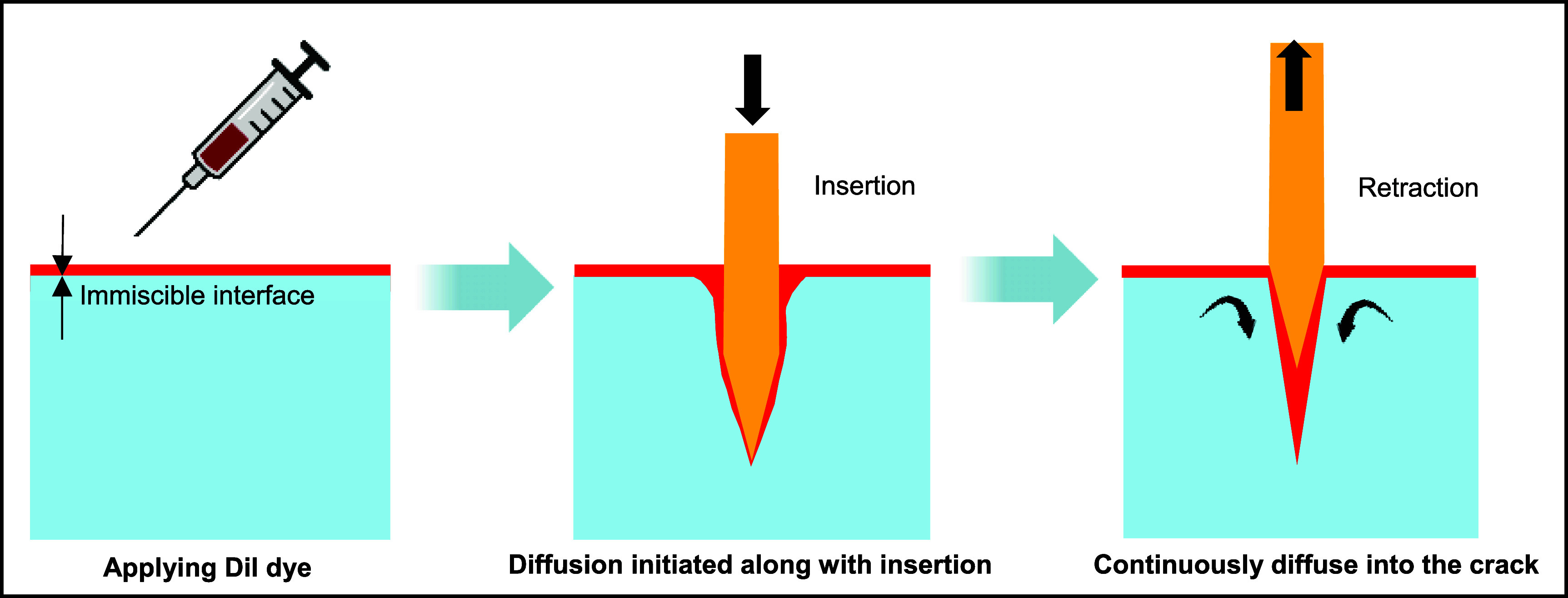
Visualization of cracks in transparent brain phantom through the diffusion of immiscible dye.

Typically, cracks produced by probes with microscale dimensions in the highly transparent hydrogel phantom were not readily visible. To address this challenge, stain diffusion was adopted to highlight the insertion-induced cracks in the hydrogel phantom, as illustrated in figure 2. The staining agent Dil dye (ThermoFisher) was prepared at a concentration of 2 mg ml^−1^ in ethanol. It was experimentally confirmed in figure S2 that the stain does not effectively diffuse into gels without cracks; instead, it selectively highlights cracks inside the gel. Furthermore, due to the nature of diffusion, a slight time delay at the second level (cf movies in SI) exists between the formation of new crack fronts and their effective detection.

### 2.5. Finite element analysis of the contact stress before puncture

Finite Element Analysis (FEA) models are developed using ABAQUS/EXPLICIT (Abaqus 2022, Dassault Systems Simulia Corp.) to examine the contact stress as probes interact with and dimple the brain phantom in the pre-puncture stage, shedding light on the stress concentration patterns at the contact area. Considering the large difference in Young’s modulus between Kapton (2.5 GPa) and the brain phantom (∼10 kPa), the Kapton probes were assumed to be rigid bodies. Four distinct probe configurations were simulated for comparative analysis: baseline ($\alpha $ = 20°, *W* = 90 *μ*m, and *T* = 25.4 *μ*m); blunt shank ($\alpha $ = 180°, *W* = 90 *μ*m, and *T* = 25.4 *μ*m); thick shank ($\alpha $ = 20°, *W* = 90 *μ*m, and *T* = 76.2 *μ*m) and a cylindrical microwire with a diameter of 50 *μ*m which summarized independently in figure S8. An isotropic hyperelastic constitutive model (Ogden model) was adopted for the hydrogel phantom, with parameters of ${\mu _1} = 1.012\,\,\,{\text{kPa}}$, ${\alpha _1} = - 16.01$, ${D_1} = 0.03\,\,\,{\text{kP}}{{\text{a}}^{ - {\text{1}}}}$ [36]. The boundary conditions are set such that the surface of the phantom in contact with the probe is free while all other surfaces of the phantom are fixed. The pre-puncture insertion process is simulated as a quasi-static process with a downward probe displacement of 100 *μ*m, which is much less than the simulated phantom thickness of 0.5 mm. It should be emphasized that the region of interest of current pre-puncture simulation focused only on the tiny contact point of the tip (∼50 *μ*m), the simulated hydrogel thickness of 500 *μ*m is relatively far (∼10 times) enough to eliminate the boundary effect, with reasonable computational cost. To optimize computation efficiency, only a quarter of the structure is simulated considering two-plane symmetry, as depicted in figure S3. The contact between the probe and the brain phantom was modeled as frictionless. The 3D hybrid linear tetrahedral elements C3D4H, which is one of the common choices for simulation of incompressible hyperelastic soft and biomaterials using Abaqus, are adopted for the brain phantom.

## 3. Results

### 3.1. Insertion force dynamics of probe dummies with different geometry

**Figure 3.**
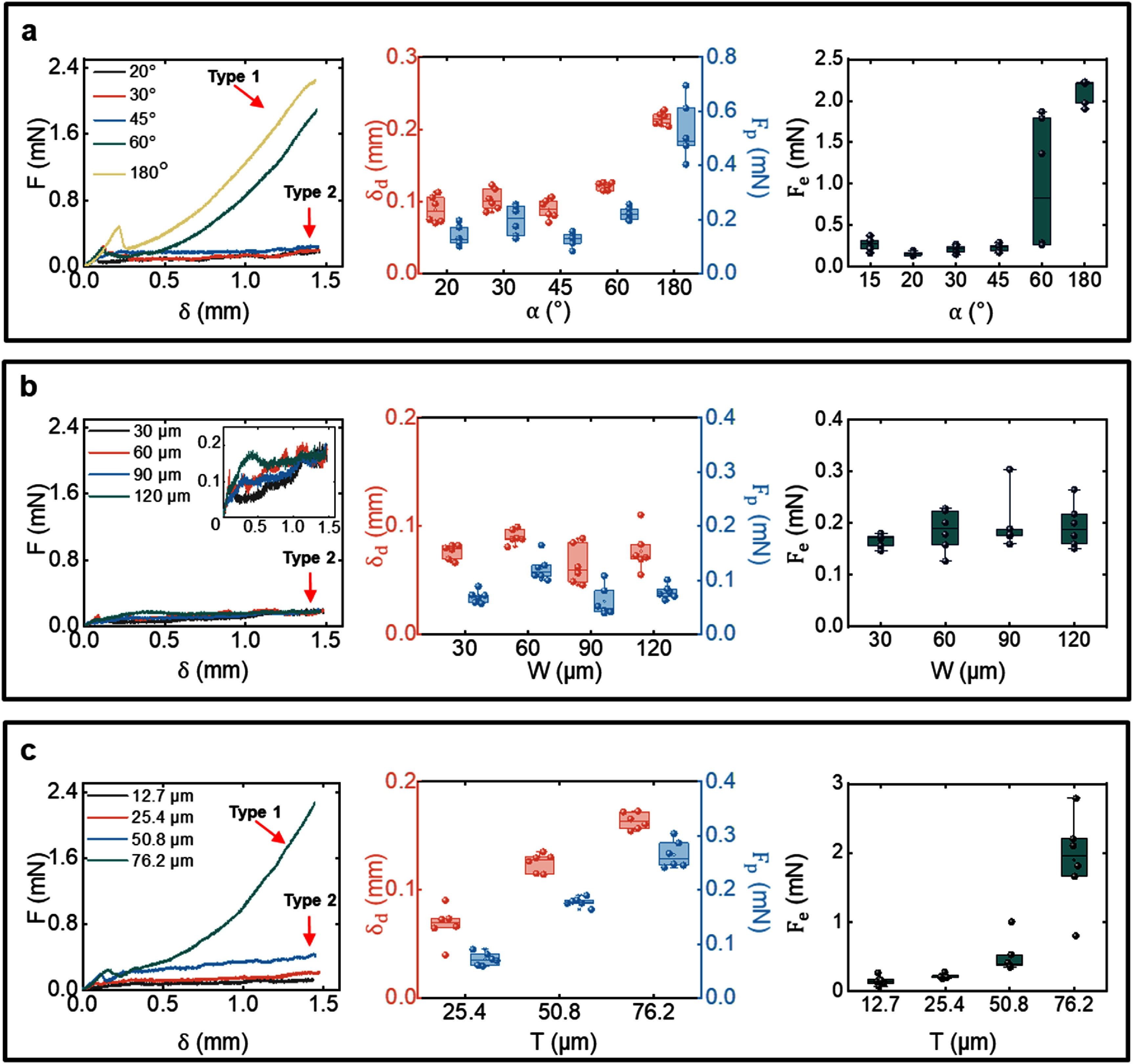
Insertion dynamics and analysis of dimpling (${{\boldsymbol{\delta}} _{\boldsymbol{d}}}$), puncture force (${{\boldsymbol{F}}_{\boldsymbol{p}}}$), and ending force (${{\boldsymbol{F}}_{\boldsymbol{e}}}$) for probes with different geometry. (a) Force dynamics versus varying tip angles, while the probe width was controlled as 90 *μ*m and thickness as 25.4 *μ*m. (b) Force dynamics versus varying widths, while the tip angle was controlled at 20° and thickness as 25.4 *μ*m. (c) Force dynamics versus varying thicknesses, while the tip angle was controlled at 20° and width as 90 *μ*m. The box plots depict the average value among six insertions for each probe configuration, with the lower and upper boundaries of the box representing the 25th percentile and 75th percentile. All data points, including outliers, are displayed in the plots.

The insertion force was measured for three groups of probe dummies with dedicated control over the shank tip angle, width, and thickness (figure 3), and six insertion tests were performed for each probe design. The representative force-displacement curves were plotted in the first columns of figures 3(a)–(c). The force-displacement curves for all six insertions of each probe design were shown in figures S4–S6. The analysis of the puncture events (dimpling ${\delta _d}$ and puncture force ${F_p}$) was summarized in the second column, and the comparison of the ending force ${F_e}$ was shown in the third column of figures 3(a)–(c).

Notably, the force-displacement responses can be clearly classified into two different types, as labeled in figure 3. Type 1 exhibited a rapidly increased force (${K_e} = 2.32 \pm 0.26{\text{ mN mm}}^{-1}$) with the ending force (${F_e} = 2.12 \pm 0.13{\text{ mN}}$) much larger than the puncture force (${F_e} = 0.53 \pm 0.10{\text{ mN}}$), as data from *α* = 180° in figure 3(a), while Type 2 featured a consistently low force with slow changes (${K_e} = 0.01 \pm 0.08{\text{ mN}}\,{\text{m}}{{\text{m}}^{ - 1}}$) with the ending force (${F_e} = 0.19 \pm 0.04{\text{ mN}}$) at the same level as the puncture force (${F_e} = 0.12 \pm 0.02{\text{ mN}}$), as data from *W* = 60 *μ*m in figure 3(b). The nature of insertion force dynamics appears to be predominantly influenced by the overall sharpness of the shank tip. For the shank geometries discussed here, the sharpness can be further delineated into two important factors linked to contact-area stress concentrations: (1) the tip angle, which was extensively studied in the literature [21, 37, 38], and (2) the longer length of the initial contact area, which is referred to as the characteristic tip width *L_c_* in this study. For the majority of probes with chisel tip, the initial contact area is a rectangle with a length equal to the thickness *T* and a much smaller width constrained by fabrication processes; hence *L_c_* = *T*. The only exception is the 180° (blunt) shank, where the initial contact area is *T* × *W* and *L_c_* is the larger of the two sizes. Based on this interpretation, the findings presented in figure 3 can be summarized as follows. First, in most cases in figures 3(a) and (b), where *L_c_* = 25.4 *μ*m, the insertion dynamics were of Type 2. Two exceptions are noteworthy: (1) the case of$\,\alpha = 60^\circ $, which appeared to represent a critical tip angle distinguishing Type 1 and 2, as both types were observed in experiments for the same probe geometry, and (2) the case of a $180^\circ $ blunt shank featuring *L_c_* = 90 *μ*m, exhibiting Type 1 dynamics. Second, figure 3(c) showed cases with $\alpha = 20^\circ $ and *L_c_* = T varying from 12.7 to 76.2 *μ*m (0.5 mil to 3 mil), Type 2 dynamics were observed for *L_c_* < 76.2 *μ*m, and the case with a critical *L_c_* = 76.2 *μ*m exhibited a mix of Types 1 and 2 dynamics. It should be emphasized that this mixed type seems to be an outlier, which was not used as a representative curve in figure 3(c) showing both features of the Type 1 and Type 2 crack mode. (cf Movie S5). After an initial straight crack (Type 2), a conical crack (Type 1) was formed at the later stage of the insertion. Reflecting on the insertion dynamics, the curve lied on between the typical Type 1 and Type 2 curve as in figure S6.

In the preliminary investigation detailed in figure S7, three different insertion speeds were utilized: 5 *μ*m s^−1^ (slow), 50 *μ*m s^−1^ (medium), and 500 *μ*m s^−1^ (fast), which were applied to both the sharp baseline probe (*α* = 20°) and the blunt probe (*α* = 180˚). Consistently, Type 2 dynamics were observed for the sharp probe, and Type 1 dynamics were observed for the blunt probe across all three insertion speeds.

For the comparison study of the blunt tungsten microwire and blunt polymer probe with similar cross-sectional areas, the results were summarized in figure S8. Both of them show the same Type 1 dynamics and very similar characterizations in the force-displacement curve.

### 3.2. Crack morphologies and actual insertion depth

**Figure 4.**
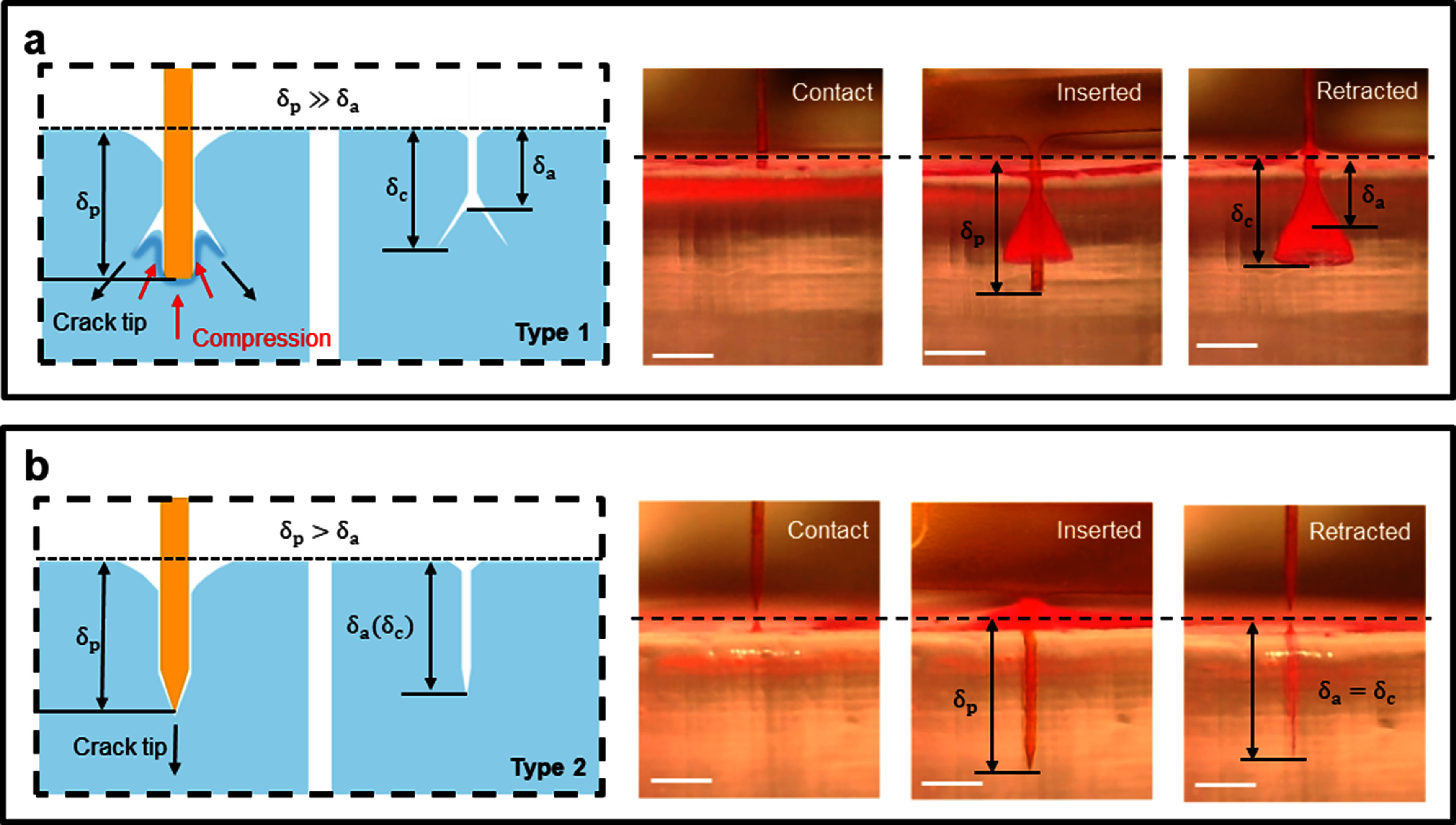
Two distinct cracking modes in hydrogel brain phantom. (a) Branched conical cracks induced by a blunt probe. (b) Straight cracks induced by a sharp probe. The snapshots illustrate probe status at various stages from experiments (see Movie S1 and S2 for more details). The notations in the illustrations: ${\delta _a}$—Actual probe penetration depth; ${\delta _p}$—post-contact probe downward displacement; ${\delta _c}$—the depth of the cracked or damaged region. Scale bar: 500 *μ*m.

The visualization technique enabled *in situ* imaging of the crack formation during the insertion process. Remarkably, all cases with Type 1 force dynamics exhibited cracks that branched into an axisymmetric conical shape, as shown in figure 4(a) and Movie S1. On the other hand, in all cases with Type 2 force dynamics, straight cracks that aligned with the insertion direction emerged, as shown in figure 4(b) and Movie S2. Additional supplementary information showcasing the branched conical crack morphology can be found in Movie S3 (*α* = 20°, *W* = 90 *μ*m, and *T* = 76.2 *μ*m) and Movie S4 (*α* = 60°, *W* = 90 *μ*m, and *T* = 25.4 *μ*m).

The *in situ* tracking of the crack formation also facilitates the measurement of the actual probe penetration depth ${\delta _a}$ which is smaller than the post-contact probe downward displacement ${\delta _p}$ (set as 1.5 mm in all tests), as illustrated in figures 4(a) and (b) for both branched and straight cracks. Additionally, the depth of the cracked or damaged region (${\delta _c}$ in figure 4) for both cracking modes can be measured and can be estimated based on the consistent observation that ${{{\delta }}_a} \le {{{\delta }}_c} \le {{{\delta }}_p}$ in all the experiments in this study. It was observed that ${\delta _a}$is clearly much smaller than ${\delta _p}$ for branched conical cracks while ${\delta _a}$is similar yet still slightly smaller than ${\delta _p}$ for straight cracks.

### 3.3. FEA analysis of stress concentration

**Figure 5.**
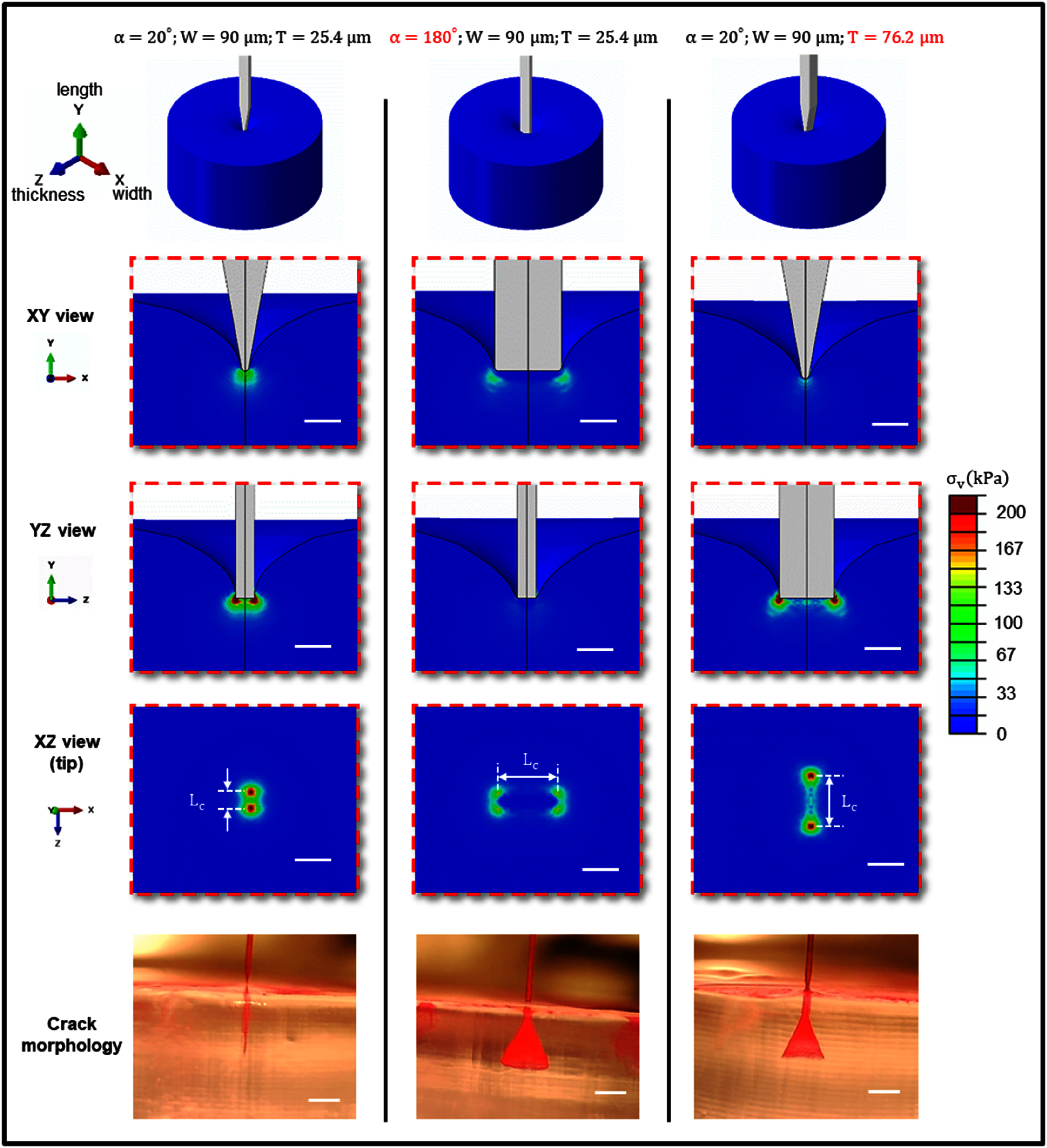
Stress concentration from FEA modeling at the pre-puncture stage. Stress concentrations are shown from various perspectives, with snapshots of the corresponding crack morphologies observed in experiments. The coordinates *X-, Y*-, and *Z*-correspond to the width, length, and thickness directions of the probe, respectively. The scale bar in the stress contour represents 50 *μ*m, whereas in the experimental images, it measures 500 *μ*m.

Three distinct probe designs were evaluated in the FEA analysis here, including the baseline design ($\alpha $ = 20°, *W*= 90 *μ*m, *T* = 25.4 *μ*m), a blunt shank design (${{\alpha }}$ = 180°, *W* = 90 *μ*m, *T* = 25.4 *μ*m), and a thick shank design ($\alpha $ = 20°, *W* = 90 *μ*m, *T* = 76.2 *μ*m). The FEA analysis of the 50 *μ*m tungsten microwire was listed independently in figure S8 as a special case study. The von Mises stress distributions were shown in figure 5 across YZ, XY, and XZ views. The stress concentration emerges at multiple locations near the edge of the contacting area throughout the entire insertion process, with patterns similar to those in figure 5 (XZ view). It should be noted that such stresses concentration effect along the circumference of implanted neural probe tip has also been reported by Muthuswamy *et al* [39] for the simulation of micromotion-induced probe-tissue interaction. Cracks dynamically propagate from locations with the highest stress concentration and exhibit two modes which is characterized by the geometric parameter—the characteristic tip width *L_c_*—that effectively reflects the sharpness of the probe tip introduced in section 3.1.

The smaller *L_c_* values (figure 5, left column) correspond to closer stress concentration sites and promote straight cracking and Type 2 dynamics featuring low insertion forces comparable to the puncture force, which suggests a series of smooth, continuous puncture events, while larger *L_c_* values increase the possibility of branched conical cracks (figure 5, middle and right columns) and Type 2 dynamics results from substantial compression within the phantom medium. As the critical sharpness factors separating the two types of dynamics are likely coupled, future experiments can be designed to determine the critical *L_c_* for each tip angle based on the specific geometry of the probes.

It should be emphasized that the current pre-puncture simulations focus only on the initial contact stress concentration. In the absence of fracture propagation mechanisms, accurately predicting post-puncture phenomena directly from FEA becomes challenging. However, envisioning the insertion process during real experiments is not complex. As the probe delves deeper and cracks propagate, stress concentration locations shift simultaneously in a highly time-dependent manner. In the case of a conical crack, two distinct stress concentration locations emerge: the point where the crack exits and the location of the probe tip. Once stress concentration reaches a certain threshold at the probe tip, a new conical crack forms from the internal interface. This dynamic behavior leads to periodic conical cracks, similar to the phenomena on a large scale reported by Muthukumar *et al* [32, 33].

Furthermore, it is noteworthy that the conical crack observed experimentally exhibits a high degree of axisymmetry, regardless of whether the testing probe possesses axisymmetry or not (cf Figure S8, comparing the non-axisymmetric shank and the axisymmetric tungsten microwire). This observation suggests that the crack propagation process appears to ‘forget’ its initial conditions, characterized by a 3D stress distribution from contact. This intriguing phenomenon warrants further investigation to gain deeper insights into the underlying mechanisms governing crack formation and propagation.

### 3.4. Mapping cracking modes through the analysis of insertion force dynamics

The two distinct types of insertion force dynamics exhibit a clear correlation with the two observed cracking modes, enabling the estimation of the damage in the phantom through the analysis of the force-displacement curves. As discussed in the previous sections, the fundamental difference between the two cracking modes is the variation in the mechanical resistance after the puncture event, leading to the introduction of a dimensionless metric:

\begin{equation*}\phi = \frac{{{K_e}}}{{{K_i}}}\end{equation*}

where ${K_i}$, ${K_e}$ are pre- and post-puncture stiffness (figure 1(b)), respectively.

**Figure 6.**
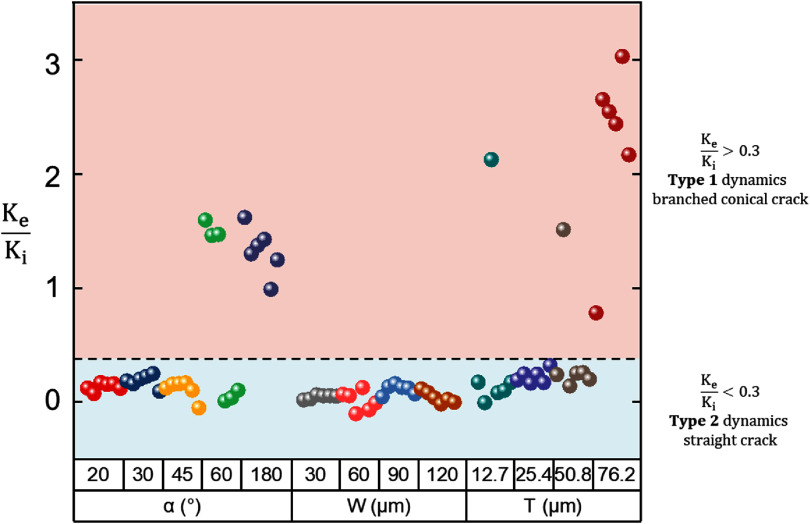
Mapping of the types of cracking induced by probes with different geometry. The branched conical cracks were observed for $\phi $ > 0.3 (light orange background area), whereas the straight cracks were observed for $\phi $ < 0.3 (light cyan background).

As illustrated in figure 6, for Type 2 dynamics that had $\phi &lt; 0.3$, straight cracking (indicated by the light cyan background in the plot) was observed, consistent with a lower fracture force required to advance a propagating straight crack compared to the initiation of a crack on an intact hydrogel surface; for Type 1 dynamics that had $\phi &gt; 0.3$, branched conical cracks emerged as indicated by light orange background in figure 6. This observation allows the indirect assessment of the crack morphology and provides an estimate of damage based on the force dynamics, *i.e.* a high $\phi $ value derived from the force-displacement curve can serve as a reliable, quantitative indicator of subpar probe sharpness that results in ineffective penetration.

## 4. Discussion

### 4.1. Effect of cracking modes on the insertion force dynamics

The insertion of a probe into a tissue-mimicking phantom comprises the pre-puncture and post-puncture stages. The pre-puncture stage is similar to an indentation process, with the measured force proportional to the stiffness of the phantom. The post-puncture puncture force can be expressed as

\begin{equation*}F = {F_{{\text{compression}}}} + {F_{{\text{friction}}}} + {F_{{\text{adhesion}}}}\end{equation*}

${F_{{\text{compression}}}}$ comes from the overall deformation of the hydrogel phantom, with forces and strain energy increasing as the probe displaces downward and releases through cracking propagation. ${F_{{\text{friction}}}}$, ${F_{{\text{adhesion}}}}$ denote friction and adhesion forces at the interface between the probe and the hydrogel, respectively.

Both ${F_{{\text{friction}}}}$ and ${F_{{\text{adhesion}}}}$ are proportional to the actual contact area($A \approx 2\left( {W + T} \right) \cdot {\delta _a}$ for the shank geometry in this study) between the probe and the phantom. Conventionally, rapid increase of the post-puncture force is attributed to ${F_{{\text{friction}}}}$ and ${F_{{\text{adhesion}}}}$, accounting for the increase in ${\delta _a}$ as the probe delves deeper. However, it overlooks possible crack branching that could lead to a substantial increase in ${F_{{\text{compression}}}}$ and is inconsistent with experimental results in two separate regards: (1) Branched conical cracks were observed to have ${\delta _a}$ much smaller than the probe displacement ${\delta _p}$, while straight cracks exhibited ${\delta _a} \approx {\delta _p}$. Therefore, the maximum interface contact area of the branched conical cracks is much smaller than that of the straight cracks, suggesting much smaller friction/adhesion forces in branched cracks. However, the total ending force (${F_e}$) exhibited an opposite comparison, contradicting the friction/adhesion models. (2) Probes with the same geometry are supposedly expected to yield similar maximum contact area and, therefore, friction/adhesion forces, yet significantly different dynamics were observed in cases with critical sharpness factors. These analyses clearly indicate a negligible contribution from the shear force in insertion dynamics and challenge conventional understanding based on friction/adhesion models.

The comparison study between the blunt polymer probe and float-tip tungsten microwire, designed to have similar cross-sectional areas as summarized in figure S8, provided additional evidence. It was revealed that adhesion/friction between the probe and the hydrogel was not the driving mechanism determining the insertion force. Despite having different friction properties, both probes exhibited remarkably similar insertion dynamics. Instead, the dominant effect was found to be the crack mode, with a very consistent Type 1 conical crack observed.

Furthermore, preliminary investigations on the effect of insertion speed (cf figure S7) revealed that crack modes were not influenced by the insertion speed. Consistent crack modes and the corresponding force dynamics of both Type 1 and Type 2 were observed across different insertion speeds. However, during slow insertion, which exhibited Type 1 dynamics with conical cracks, a smaller ending force was observed compared to medium and fast insertions. This difference presumably stemmed from a mismatch between the crack tip propagation speed and the probe tip speed in Type 1 dynamics. During slow insertion, the probe was inserted concurrently with the propagation of the conical crack tip. As a result, the difference between the probe’s downward displacement (${\delta _p}$) and the depth of the crack (${\delta _c}$) became smaller compared to the situation of fast insertion, leading to a smaller internal compression force, as observed.

### 4.2. Estimating damage and actual insertion depth through insertion force dynamics analysis

For transparent phantoms, the novel visualization technique developed here allows for *in situ* assessment of insertion-induced cracks and damage. The depth measurements on ${\delta _a}$ and ${\delta _c}$ provided superior metrics for the actual penetration depth and phantom damage, respectively, representing a significant advantage over conventional understanding that associate ${\delta _p}$ with both the insertion depth and damage depth, regardless of the cracking mode.

For opaque *in vivo* tissues consisting of diverse layers and cells, direct visualization of damage and fractures during probe insertion remains challenging. Conventional histological methods [40], which involve intricate post-processing steps such as perfusion and fixation, may introduce undesirable damage that alters the original insertion-induced cracks. This study unveiled a clear and reliable correlation between cracking modes and the insertion dynamics, suggesting the possibility of inferring tissue damages by analyzing force-displacement responses: Type 1 dynamics implies the presence of a branched conical cracking region and large compression force that could lead to further complications; Type 2 dynamics suggests a confined, minimum damaged region, with the cross-section equal to the footprint of the probe, and a depth (${\delta _a} \approx {\delta _c} \approx {\delta _p}$) that can be directly extracted from the force-displacement curve. While direct and accurate assessments of damages from systematic biological studies are lacking, the insights derived from this study present an effective and valuable tool for indirect estimates of the damage and actual insertion depth.

### 4.3. Suitability of hydrogels as brain phantoms for probe insertion tests

The suitability of hydrogel brain phantoms for studying neural probe insertion dynamics remains a subject of debate due to confusing interpretations of force dynamics. In a study by Obaid *et al* [20, 41], *in vivo* insertion tests showed near-zero force, while phantom studies displayed a gradually increasing trend with multiple large force peaks. It was hypothesized that a sequence of friction-driven stick-slip events was the cause of such force dynamics. However, the Type 1 dynamics observed in both *ex vivo* tissue and hydrogel phantom tests indicate the formation of branched conical cracks in both media. Insights from the current study suggest that these reported discrepancies in force dynamics are likely the consequences of the two distinct cracking modes. Given that the shank geometry remained consistent between the sets of reported studies being compared, the variation in the insertion dynamics should be attributed to the difference in fracture characteristics between the *in vivo* tissues, *ex vivo* tissues, and the phantom. Large insertion forces (Type 1 dynamics) observed in phantom or *ex vivo* studies can be inconsistent with insertion dynamics during *in vivo* insertion tests when there is a discrepancy in the fracture properties between the different sets of studies. Addressing this discrepancy in fracture mechanics characteristics when selecting phantom materials is crucial for ensuring that phantom tests hold meaningful implications for force dynamics during *in vivo* insertions. It should be noted that the common practice that adopts the peak insertion force observed in phantom studies as the sole factor for the optimization of the probe geometry [42] is unreliable unless phantoms with both fracture and stiffness characteristics matched to the *in vivo* tissues are utilized. Nevertheless, despite potential shortcomings, hydrogel brain phantoms are still valuable brain mimics for studying the rigidity/stability of probes and the effective sharpness of the probe. It should be acknowledged that direct experimental evidence of unique crack modes is limited to the hydrogel brain phantom in the current study. For real tissue, further investigation to validate some of the hypotheses discussed is needed from future research.

Beyond probe insertion tests, the identified cracking mechanisms should also be considered in studies utilizing dye-staining techniques in hydrogel phantoms to simulate drug diffusion properties in tissues. This is especially relevant for investigations involving penetrating probes with microfluidic channels. For instance, Mu *et al* [43] reported dye-stained patterns resembling a conical shape, interpreting the phenomenon as strain-driven directional diffusion into the hydrogel medium without examining the possibility of branched conical crack formation. In future hydrogel phantom studies involving probe penetrations, it is advisable to incorporate experimental designs that account for the cracking mechanisms to identify the origin of such dye-stained patterns.

## 5. Conclusion

In this study, the insertion force dynamics of polymer probe dummies with different geometry in brain phantom (0.6% w/w agarose hydrogel) is systematically studied by *in situ* visualization of the crack formation and force dynamics recording. Two distinct cracking mechanisms—branched conical cracks and straight cracks—were observed, each resulting in a unique type of force dynamics. Blunt probes lead to branched conical cracks and Type 1 dynamics featuring large post-puncture forces stemming from mechanical resistance at the blunt, conical crack tip. Sharp probes induce straight cracks and Type 2 dynamics with consistently low forces at the level of the puncture force. These findings challenge conventional interpretations of the force dynamics based on friction/adhesion models.

Confirmed by 3D FEA that reveals pre-puncture contact stress concentration patterns, the probe’s tip angle and characteristic length were identified as two critical factors that affect the effective sharpness of the probe. Experimental phantom insertion tests showed that probe configurations with a tip angle smaller than 60° or a characteristic tip width less than 76.2 *μ*m predominantly exhibited straight cracks in the hydrogel, while blunter probes led to branched conical cracks.

Interpretations and novel methodologies reported here contribute to a better understanding of standard insertion testing with brain phantoms. These insights can be extended to study rigid probes (*e.g.* commonly used silicon or metal probes) or probes with other geometries. To accurately simulate the force dynamics during *in vivo* insertion via phantom studies, it is crucial to carefully consider tissue mimics with not only similarity in stiffness properties but also in fracture properties when conducting insertion tests. Nevertheless, hydrogel phantoms approximating only the stiffness of *in vivo* tissues remain suitable for studying the stiffness/stability and effective sharpness of probe design, provided that experiments and analyses incorporate insights from this study. Lastly, this study provides an effective way for estimating tissue damage resulting from penetrating devices based on force dynamics analysis and cautions against the interpretation of the force-displacement responses or dye-stained patterns in hydrogels without carefully accounting for the cracking mechanisms in insertion tests.

## Data Availability

All data that support the findings of this study are included within the article (and any supplementary files).
